# Design and analysis of statistical probability distribution and non-parametric trend analysis for reference evapotranspiration

**DOI:** 10.7717/peerj.11597

**Published:** 2021-06-18

**Authors:** Sajid Gul, Jingli Ren, Neal Xiong, Muhammad Asif Khan

**Affiliations:** 1 Henan Academy of Big Data, Zhengzhou University, Zhengzhou, Henan, China; 2 School of Mathematics and Statistics, Zhengzhou University, Zhengzhou, Henan, China; 3 Departments of Mathematics and Computer Sciences, Northeastern State University, OK, United States of America; 4 School of Statistics, Jiangxi University of Finance and Economics, Nanchang, Jiangxi, China; 5 Department of Statistics, Islamia College University, Peshawar, Khyber Pakhtunkhwa, Pakistan

**Keywords:** Anderson darling, Irrigation, Khyber Pakhtunkhwa, Kolmogorove–Smirnov, Seasonal

## Abstract

Accurate estimates of reference evapotranspiration are critical for water-resource management strategies such as irrigation scheduling and operation. Therefore, knowledge of events such as spatial and temporal reference evapotranspiration (ET_o_) and their related principle of statistical probability theory plays a vital role in amplifying sustainable irrigation planning. Spatiotemporal statistical probability distribution and its associated trends have not yet has explored in Pakistan. In this study, we have two objectives: (1) to determine the most appropriate statistical probability distribution that better describes ET_o_ on mean monthly and seasons wise estimates for the design of irrigation system in Khyber Pakhtunkhwa, and (2) to check the trends in ET_o_ on a monthly, seasonal, and annual basis. To check the ET_o_ trends, we used the modified version of the Mann-Kendall and Sen Slope. We used Bayesian Kriging for spatial interpolation and propose a practical approach to the design and study of statistical probability distributions for the irrigation system and water supplies management. Also, the scheme preeminent explains ET_o_, on a monthly and seasonal basis. The statistical distribution that showed the best fit ET_o_ result occupying 58*.*33% and 25% performance for the design of irrigation scheme in the entire study region on the monthly level was Johnson SB and Generalized Pareto, respectively. However, according to the Anderson-Darling (AD) and Kolmogorov–Smirnov (KS) goodness of fit measure, seasonal ET_o_ estimates were preferably suited to the Burr, Johnson SB & Generalized Extreme Value. More research work must be conduct to assess the significance of this study to other fields. In conclusion, these findings might be helpful for water resource management and policymaker in future operations.

## Introduction

Reference evapotranspiration is one of the most crucial functions in the Hydrological progression, and the climate is affected by this phenomenon (Allen et al., 1998). The term evapotranspiration involves evaporation from the soil, and plant surfaces include transpiration from plant leaves. Evaporation involves converting liquid water into water vapor and removing it from evaporation’s surface (Allen et al., 1998). Recently, water crisis management planning, agriculture water management, and water resource projects were scheduled based on ET_o_. An estimate of irrigation water requirement is vital for water planning and operation of a sophisticated irrigation system worldwide. Therefore, ET_o_ estimates have frequently been using in hydrology, agriculture water management, irrigation, drainage engineering designing, water crisis supervision, scheduling, and operational supervision (Valipour, 2014a; Valipour, 2014b; Valipour, 2015b; Valipour, 2015c).

Besides, several researchers showed that an estimate of irrigation water requirement is significant for the function of an intricate irrigation system in many parts of the world (Tabari, Grismer & Trajkovic, 2013; Tabari & Talaee, 2011; Thepadia & Martinez, 2012). An accurate estimation of the reference or crop evapotranspiration (ET_*o*_, ET_*c*_) can utilize the accessible water resources such as stored water for the arid period. To estimate ET_*c*_ in the first stage, ET_o_ is calculated and then multiplied by the harvest coefficient (K_C_) (Allen et al., 1998; Doorenbos, Pruitt & Aboukhaled, 1997).

Further, Allen et al. (1998) define crop evapotranspiration as an evapotranspiration rate with an inferred crop height from the hypothetical crop and a fixed resistance to the canopy (70 s per meter). The albedo would cautiously acknowledge the proximity to evapotranspiration from a widespread green grass surface of equal height, aggressive growth, and strictly shading the soil. Likewise, many well-known researchers discussed and strongly suggested the FAO-56 (PM) technique as a standard procedure for accurate assessment and estimation of ET_o_, if lysimeter data is unavailable or limited on reference evapotranspiration (Gavilán et al., 2006; Irmak, Allen & Whitty, 2003; Nandagiri & Kovoor, 2006; Trajkovic, 2007; Utset et al., 2004).

On the other hand, numerous studies have shown that the temperature-based Hargreaves-Samani formula could provide accurate measures of ET_o_ for five or longer days in various corners of the world (Droogers & Allen, 2002; Hargreaves & Allen, 2003; Jensen et al., 1997; Landeras, Ortiz-Barredo & López, 2008; Martí et al., 2015). Ultimately, the Hargreaves-Samani system’s implied when partial data such as relative humidity, solar radiation, and wind speed is sufficient. Extensive research has been carried out in different parts of the world for estimating ET_o_ and comparison models by using limited climatic data. They compared the Makkink, Turc, Hargreaves, and Priestley–Taylor methodologies for estimation. The studies revealed that the Radiation-based approach was more precise and proper than the Hargreaves method (Bois et al., 2008; Tabari, Grismer & Trajkovic, 2013; Valipour, 2015a; Xu & Singh, 2002). However, some researchers also observed that the suggested ET_o_ values showed overestimation in damp conditions (Itenfisu et al., 2003; Temesgen et al., 2005; Trajkovic, 2005; Valipour, 2017). Similarly, several research studies also showed underestimated ET_o_ in arid and semi-arid regions in diverse locations (Azhar & Perera, 2011; Benli et al., 2010; Droogers & Allen, 2002; Khoob, 2008). Consequently, in arid and semi-arid environments, local calibration and justification of such concepts are considerable (Da Cunha, Magalhaes & De Castro, 2013).

Moreover, several research findings associated with environmental events have required also evaluating probability distributions and improved clustering procedure based on energy consumption, prediction, and risk assessment in networks (Blain, 2012; Blain & Meschiatti, 2014; Huang et al., 2017; Nam & Choi, 2014; Rahman et al., 2013; Wu, Xiong & Wu, 2017; Zhang et al., 2015). As a result, the elementary probability theory of extreme events has been developing and used extensively. Thus, extreme events’ statistical probability distribution modeling remains of great concern and plays a vital part in water resource supervision in different conditions (Katz, 2010).

In contrast, climate change has been detected not only in individual parameters such as temperature and precipitation but also in integrated parameters such as ET_o_ (Ma et al., 2017). The most influential factor for climate change and the water cycle is evapotranspiration, as it is the only linkage between the water balance and the surface energy balance of the land (Wang et al., 2014). Some well-known authors worldwide suggested that knowledge about best fit statistical probability model in site ease is a correct hydrological prediction in the dimensioning drainage and irrigation planning (Fernandes, Wolff & Folegatti, 2019; Nam & Choi, 2014; Uliana et al., 2017; Yoo, Choi & Jang, 2008).

Besides, climate change is almost proven globally; the pattern of evapotranspiration is not apparent. ET_o_ trends can increase or decrease depending on climatic conditions and regions. However, shifts in hydrological cycle components, such as ET_o_, display multiple increasing and reducing patterns (Donohue, McVicar & Roderick, 2010; Gao et al., 2006; Liu et al., 2010; Shenbin, Yunfeng & Thomas, 2006; Xu et al., 2006; Zhang et al., 2009). Also, numerous researches in several parts of the globe have shown a decline in evapotranspiration over the past decades. However, some have identified the contrary phenomenon, which is a rise in the trend of evapotranspiration (Croitoru et al., 2013; Mohsin & Lone, 2020; Pandey & Pandey, 2013; Shan et al., 2015; Shi et al., 2017; Talaee, Some’e & Ardakani, 2014; Vicente-Serrano et al., 2014a; Vicente-Serrano et al., 2014b; Wang et al., 2014). Therefore, most of the works conducted to identify ET_o_ trend fluctuations checked by the traditional non-parametric Mann-Kendall and Sen Slope estimator tools. In the meantime, those experiments have not measured the effect of coefficients of autocorrelation with different lags. However, only in specific studies is the first order (lag-1) autocorrelation regarded (Amirataee, Montaseri & Sanikhani, 2016; Palizdan et al., 2014; Sayemuzzaman, Jha & Mekonnen, 2015; Tabari, Nikbakht & Talaee, 2012). Consequently, it may be crucial to exclude autocorrelation’s effect to ascertain the changes to reference evapotranspiration.

Hence, in the present study, we use the updated version of the Mann-Kendall (Hamed & Rao, 1998) to check monthly, seasonal, and annual ET_o_ pattern variations in the Khyber Pakhtunkhwa. Therefore, it is essential to note reference evapotranspiration patterns and their position in regional dry and wet environments. It could provide a scientific foundation for managing and distributing regional water resources and technical judgment on flood and drought disaster prevention (Ma et al., 2017).

In South Asia, Pakistan is situated at the intersection of Central Asia and the Middle East, enabling its place of tremendous value in the world with the most incredible scenery, stretching from the Arabian Sea, its southern boundary, to the stunning and highest mountain ranges of the Himalayas-Karakoram-Hindukush (HKH) in the north, also recognized as the third pole of the world (Yao et al., 2012). According to the latest census estimate, its total population is (207 774 520) (Statistics, 2017). On the other hand, many working classes are related either directly or indirectly to the agriculture sector (Rehman & Chandio, 2019; Rehman et al., 2015; Rehman et al., 2016).

Khyber Pakhtunkhwa (KP) is one of the four Pakistani provinces situated in the state’s northwestern areas. KP is positioned predominantly on the Iranian plateau and includes the intersection where the slopes of the Hindu Kush Mountains approach the Indus-watered hills in South Central Asia on the Eurasian plate. The economy is agricultural in Khyber Pakhtunkhwa; therefore, irrigation is about one-third of the farming area. The legendary Khyber Pass links the province to Afghanistan, while Circle Bakote Abbottabad Kohala Bridge is a central crossing point in the east over the Jhelum River. Kabul, Swat, Chitral, Kunhar, Siran, Panjgora, Bara, Kurram, Haroo, Gomal, and Zhob are the main basins that traverse the province. The province could be classified physically into two zones, including the northern one extending from the Hindu Kush range to the Peshawar basin border and the southern one extending from Peshawar to the Derajat basin. The north area is cold and snowy with heavy rainfall in winters and good summers with the Peshawar basin exemption, hot in summer, and cold in winter with the Peshawar basin exemption.

In this present research paper, the two practical approaches, like Kolmogorov–Smirnov and Anderson-Darling test, are used to propose statistical probability distributions for the irrigation system and water resource management. This highlights and explores the probability distribution that best fits and describes the reference evapotranspiration ET_o_ monthly and the seasonal basis using the most appropriate estimation method FAO-56(PM) and temperature-based estimation equation Hargreaves-Samani ET_o_ for our selected study area known as Khyber Pakhtunkhwa, Pakistan. Focusing on irrigation dimension and considering the water resources’ significance and the need for fair water use in agriculture planning in the study region. The reference evapotranspiration trends analysis has done by using modified Mann-Kendall (Hamed & Rao, 1998) and the Sen Slope estimator on a monthly, seasonal, and annual time basis. The rest of this paper is a brief description of the study area, the statistical probability distributions, and the methods used to estimate evapotranspiration in ‘Methods and Material’. ‘Selection Criteria for Statistical Probability Distribution’ describes selected locations’ results and a brief description of the goodness of fit and Statistical probability distribution selection criteria. The results and discussion have described in ‘Results and Discussion’. The summary and conclusions have discussed in ‘Summary and Conclusion’.

## Methods and Material

### Site description and meteorological data

**Figure 1:**
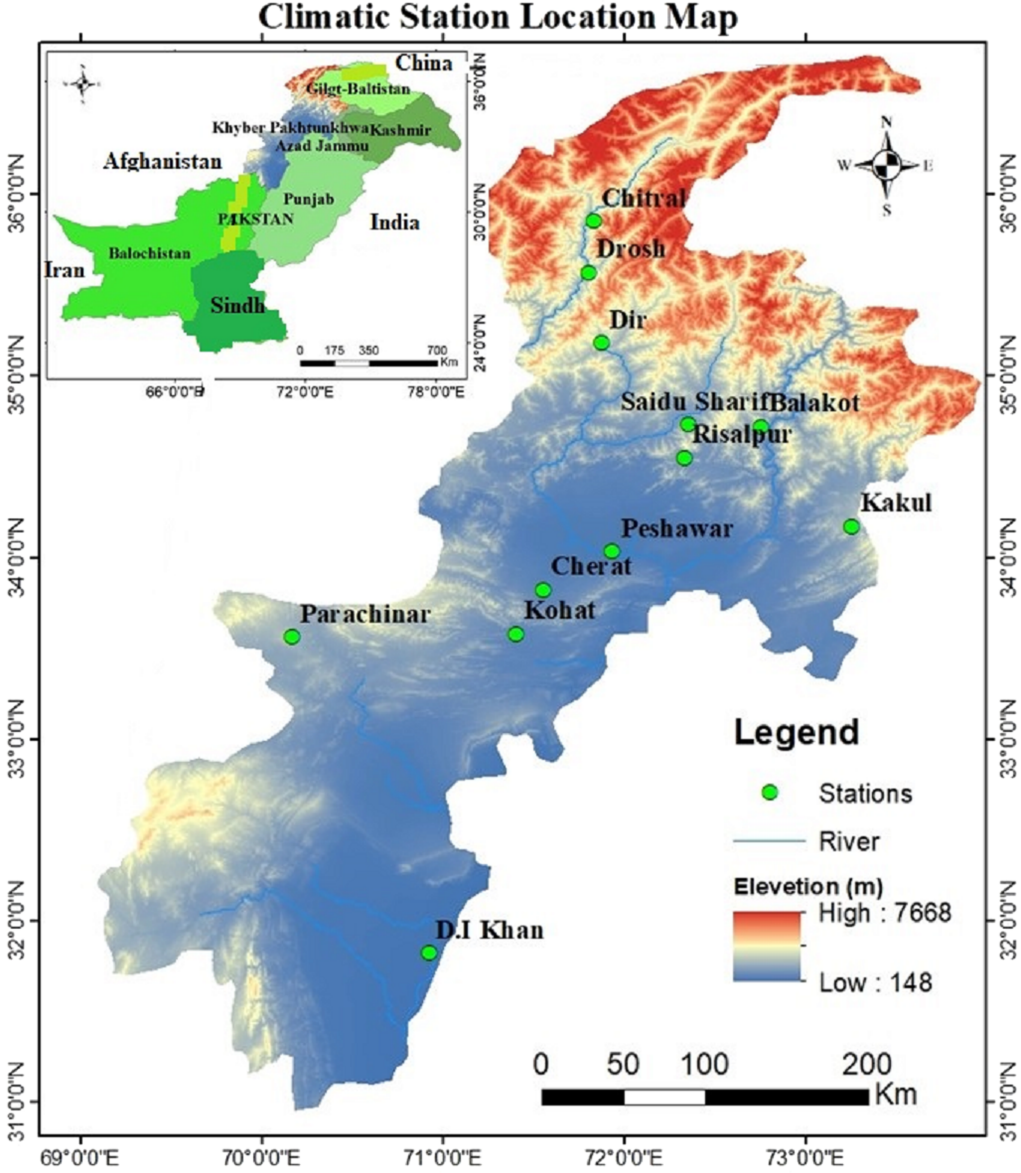
Terrestrial visualization of selected stations of Khyber Pakhtunkhwa province of Pakistan.

Khyber Pakhtunkhwa has a territory of 101*,* 741 km^2^ with about approximately 36 million population, making KP the third largest provincial economy of Pakistan. The entire region varies based on topography, from the north’s green fields to the south’s dry, rocky zones. With profoundly warm summers to cold winters, the climate can be extreme. Despite these weather extremes, agriculture in the study area matters much and feasible. Due to its diverse agroecological diversity, KP is especially vulnerable to the effects of climate change (Gul et al., 2018). In this research study, twelve climate stations distributed in the Khyber Pakhtunkhwa zone has appointed for our exploration. The cities concerned are Balakot, Cherat, Chitral, Kakul, Parachinar, Dir, Drosh, DI Khan, Kohat, Peshawar, Risalpur, and Saidu Sharif (Gul et al., 2020). A set of average monthly data collection of wind speed, air temperature, relative humidity and precipitation at these sites under standard grass height. We used Pakistan Meteorological Department (PMD) well-irrigated climate data to obtain the FAO-56(PM) and Hargreaves-Samani dependent ET_o_ values from twelve centers covering the period from 2000 to 2009. The overview of the various climate stations is presented in Table 1 and illustrated graphically in Fig. 1.

### Methodology for proposed analysis scheme

This analysis used a ten-year data set of reference evapotranspiration estimates using the FAO-56(PM) and Hargreaves-Samani (HS) estimation methods. Also, the estimated ET_o_ adjusted for Twenty-one Statistical probability distribution, as follow Burr, Johnson SB, Generalized Extreme Value (GEV), Log-Pearson-3(LP3), Normal, Logistic, Burr(4P), Weibull(3P), Generalized Extreme Value(4P), Gamma, Lognormal-3P (LN(3P)), Gumbel Min, Gen. Gamma, Gamma (3P), Cauchy, Lognormal, Weibull, Rayleigh (2P), Gumbel Max, Rayleigh and Generalized Pareto. Moreover, we used the Maximum Goodness method to adjust the best fit selected probability distributions through Kolmogorov–Smirnov and Anderson-Darling goodness of fit statistic. For this purpose, the maximum likelihood (MLE) has used the Bayesian Information Criteria (BIC) and Akaike Information Criteria (AIC) to estimate the adapted probability distribution parameters. Therefore, to contribute to all studied regions and attain parameter values, the required distribution, which provided excellent adjustment to most ET_o_ data, was the most appropriate and picked. We used Kolmogorov–Smirnov and Anderson-Darling goodness of fit statistics to confirm whether the distribution used for all the data series conformed to the data.

**Table 1:** Descriptive statistics during the entire study period.

**Stations**	**N**	**Min**	**Max**	**Mean**		**SD**
Statistics		Statistic	Statistic	Statistic	SE	Statistic
Balakot	120	0.99	7.90	3.45	0.16	1.72
Cherat	120	0.52	8.34	3.92	0.15	1.70
Chitral	120	0.71	12.60	4.71	0.29	3.22
DI Khan	120	1.14	8.94	4.01	0.18	1.99
Dir	120	1.05	9.63	4.27	0.21	2.31
Drosh	120	0.71	11.55	4.42	0.25	2.73
Kakul	120	0.82	7.26	3.03	0.14	1.49
Kohat	120	1.14	13.07	4.88	0.24	2.65
Parachinar	120	0.40	9.10	3.35	0.19	2.03
Peshawar	120	1.08	11.19	4.51	0.25	2.77
Risalpur	120	0.89	50.02	6.08	0.71	7.49
Saidu Sharif	120	1.05	9.63	4.27	0.21	2.31

Furthermore, we have used two data analysis tools such as MS Excel and Evapotranspiration packages, an open-source R (R.3.6.1) statistical software program for computation of FAO-56(PM) and Hargreaves-Samani methodologies. Also, for adjusting different distribution models in Non-censored or Censored data, the fit “Distrplus” package was used in the statistical software package (R 3.6.1) and R Studio. In this context, we used Arc GIS software for generating the spatial distribution maps, while Bayesian Kriging strategies have been used for interpolation purposes. The methodologies are discussed step by step in the following sub-sections.

### Computation models for reference evapotranspiration

#### Penman-Montieth equation

The Food and Agriculture Organization (FAO) of the United Nations and ASCE have advised the Penman-Montieth equation (American Society of Civil Engineers) as the single and standard technique to estimate reference evapotranspiration and to assess other equations in a situation where the appropriate information is available (Allen et al., 1998). This strategy is physically dependent and can usually be enforced without any further alteration of input parameters (Gong et al., 2006). The FAO-56(PM) standard scheme (Allen et al., 1998) has been used in the current study to measure ETo for the recent data analysis;

(1) \begin{eqnarray*}E{T}_{o,PM}= \frac{0.408\Delta \left( {R}_{n}-G \right) +\gamma \left( \frac{900}{T+273} \right) {U}_{2} \left( {e}_{s}-{e}_{a} \right) }{\Delta +\gamma \left( 1+0.34{U}_{2} \right) } \end{eqnarray*}

where

*ET*_*o*,*PM*_ = is Reference Evapotranspiration estimated by FAO-56(PM) [mm day^−1^].

*R*_*n*_ = is net radiation [MJ m^−2^ day^−1^], *G* = is soil heat flux [MJ m^−2^ day^−1^].

*γ* = is the Psychometric constant [kPa (^o^C^−1^)], *e*_*s*_ = is the saturation vapor pressure [kPa].

*e*_*a*_ = is the actual vapor pressure [kPa].

Δ = is the slope of the saturation vapor pressure-temperature curve [kPa (^o^C^−1^)].

*T* = is the daily air temperature [^o^C], *U*_2_ = is the wind speed at 2 m hight [ms^−1^].

Allen et al. (1998) known as the Penman-Monteith approach, proposed a complete collection of equations according to the available climate data set and time phase calculation.

#### Hargreaves-Samani equation

The Hargreaves and Samani (1985) equation is a famous symbolize edition to estimates ET_o_ values based on temperature (Hargreaves & Allen, 2003). The Hargreaves-Samani procedure is theoretically comparable editions, too Hargreaves & Samani (1982). The Hargreaves-Samani process intended to make the earlier editions transparent by reducing the number of measured air temperature data and using extraterrestrial radiation (Ra) as a replacement for sunlight or radiation data (Hargreaves & Allen, 2003). Therefore, the FAO adopted the Hargreaves-Samani estimation method for ETo estimation, whereas air temperature alone is merely accessible (Allen et al., 1998; Hargreaves & Allen, 2003). The mathematical form of the Hargreaves-Samani equation given by Allen et al. (1998):

(2) \begin{eqnarray*}E{T}_{HS}=0.0023{ \left( {T}_{max}-{T}_{min} \right) }^{0.5} \left( Tmean+17.8 \right) {R}_{a}\end{eqnarray*}

Where;

*ET*_*HS*_ =ET estimated by using the above HS equation (mm per day)

*R*_*a*_ =extraterrestrial radiation (mm per day)

*T*_*mean*_ = mean air temperature in degree Celsius

*T*_*max*_ = daily maximum temperature in degree Celsius

*T*_*min*_ = daily minimum temperature in degree Celsius

## Selection Criteria for Statistical Probability Distribution

In this paper, our projected scheme for the design and analysis of statistical probability distribution for an irrigation system and water resource management tries to discover the related logic based on the appropriate test procedure’s goodness. The process to determine whether a sample of n observations (x_1,_x_2,…_x_n_) can be considered a sample from a given specified distribution or not is known as the Goodness-of-fit test.

There is a wide range of tests available in the literature to determine whether a sample could have been drawn from a specific distribution. The test are Kolmogorov–Smirnov (KS), Anderson-Darling (AD), Chi-sq (*χ*2 test), Shapiro–Wilk (SW), Hosmer–Lemeshow (HL), and Henze-Zirkler (HZ). However, in the current investigation, the Kolmogorov–Smirnov and Anderson-Darling are used to select probability distributions. The KS and AD tests’ principal advantage is their ability to detect variations in the probability distribution’s overall shape because the test can detect differences across all scales (Darling, 1957). Thus, it is also suitable for small samples (Pettitt, 1976). Strength in the area of engineering is that For that purpose, both KS and AD are appropriate in the following perspectives: (1) when disparities are apparent, but normality is maintained; (2) when the sample size is small; (3) when variances are similar but not symmetrical; (4) when shift between two distributions does not affect only the upper or lower extremities.

In essence, we calculate the “distance” between the sample of experience data and the distribution we are examining, such as test statistics. We then measure the distance to a specific threshold value, such as a critical value. Only if the test statistic is smaller than the critical value with the significance amount (alpha = 0.05) can the configured probability distribution be considered a good fit. Since the statistics on the goodness-of-fit test describe the distance between the data and the distributions fitted. Thus, the distribution with the lowest statistical value is the one that best matches the distribution of probability. Moreover, the top three out of twenty-one distributions are taken for every single station.

The AD test also has additional benefits over the KS test. First of all, the distribution tails are susceptible to differences. Secondly, the AD test is more effective, even in large sample sizes, in detecting minimum differences (Engmann & Cousineau, 2011). Therefore, the top two probability distributions were selected for the whole study area, according to the Anderson-Darling(AD) goodness-of-fit test.

### Kolmogorov–Smirnov (KS) test

The Kolmogorov–Smirnov (KS) framework was created in the early 1930s by Kolmogorov (1933) and continued by Smirnov (1939) as a method for detecting differences in hypothetical distributions. The KS statistic for a cumulative distribution H(x) for a given data set of x data is

(3) \begin{eqnarray*}K{S}_{n}= \left( \sqrt{n} \right) su{p}_{x} \left\vert {H}_{n} \left( x \right) -H \left( x \right) \right\vert ,\end{eqnarray*}

H(x) will be rejected if *KS*_*n*_ is larger than the critical value *KS*_*α*_ at (alpha = 0.01, 0.05, and 0.10). If the observed distribution exceeds the theoretical expectations, the null hypothesis will be rejected. For more detail, see this article (Massey Jr, 1951).

### Anderson-Darling (AD) test

This test was developed by Anderson & Darling (1952) as a new statistical test for detecting sample distribution departures from normality. Assuming that H is the observed distribution and Hn is the function of the observed (sample) function, then the quadratic function of the differences between H and Hn is given by the empirical function (Stephens, 1974). Quadratic EDF statistics is.

(4) \begin{eqnarray*}n\int \nolimits \nolimits _{-\infty }^{\infty }{ \left( {H}_{n} \left( x \right) -H \left( x \right) \right) }^{2}w \left( x \right) dH \left( x \right) \end{eqnarray*}

where the *w(x)* is a weight function,

The Anderson & Darling (1954) test is based on the distance

(5) \begin{eqnarray*}A=n\,\int \nolimits \nolimits _{-\infty }^{\infty } \frac{{ \left( {H}_{n} \left( x \right) -H \left( x \right) \right) }^{2}}{H \left( x \right) \left( 1-H \left( x \right) \right) } dH \left( x \right) \end{eqnarray*}

Which is obtained when the weight function is *w(x)* = [*H* (*x*) (1 –*H* (*x*))]^−1^, with which AD distance places more weight on observations in the tails of the distribution. The AD test is defined as the null hypothesis (H_o_): The data follow a specific distribution versus the Alternative statement H_a_: The data do not follow the specific distribution. We can assess whether the observed data sample x_i_(i = 1, 2, 3, n) comes from some specified probability distribution. The test indicates that with a given underlying distribution, and assuming that the data is drawn from this distribution is coherent with it or not.

### Statistical non-parametric trend analysis

#### The Modified Mann-Kendall test

The Mann-Kendall (Kendall, 1975; Mann, 1945) test is the most commonly utilized non-parametric tool for assessing dynamics in the data analysis of meteorological time series. The null hypothesis is in the MK test; the knowledge is independent and ordered at random. However, autocorrelation (positive) within the data seeks to increase the probability of trend detection when there is no trend in reality and conversely. After all, it is generally a well-known phenomenon that some researchers have addressed this issue by ignoring the autocorrelation consequence. A modified form of the MK test (Hamed & Rao, 1998) has been used in the current study. The benefit of using the modified MK test is that the evidence of autocorrelation is robust. Adjusted variance (Var(S)) is used in this method to determine Z statistics from the standard Mann-Kendall test (Hamed & Rao, 1998). The mathematical form of the MK test is first determined using the S_mk_ statistics given below.

(6) \begin{eqnarray*}{S}_{mk}& =\sum _{i=1}^{n-1}\sum _{j=i+1}^{n}sgn \left( {x}_{j}-{x}_{i} \right) \end{eqnarray*}

(7) \begin{eqnarray*}sgn \left( {x}_{j}-{x}_{i} \right) & = \left\{ \begin{array}{@{}l@{}} \displaystyle 1~~~~~if~{x}_{j}\gt {x}_{i} \\ \displaystyle 0~~~~~if~{x}_{j}={x}_{i} \\ \displaystyle -1~~~~~if~{x}_{j}\lt {x}_{i} \end{array} \right. \end{eqnarray*}

(8) \begin{eqnarray*}E \left( {S}_{mk} \right) & =0\end{eqnarray*}

(9) \begin{eqnarray*}Var \left( {S}_{mk} \right) & = \frac{n \left( n-1 \right) \left( 2n+5 \right) -{\mathop{\sum \nolimits }\nolimits }_{i=1}^{p}{t}_{i} \left( {t}_{i}-1 \right) \left( 2{t}_{i}+5 \right) }{18} \end{eqnarray*}

(10) \begin{eqnarray*}Var \left( {S}_{mmk} \right) & =Var \left( {S}_{mk} \right) \ast W\end{eqnarray*}

(11) \begin{eqnarray*}W& =1+ \left( \frac{2}{n \left( n-1 \right) (n-2)} \right) \ast \sum _{k=1}^{n-1} \left( n-k \right) \left( n-k-1 \right) \left( n-k-2 \right) \ast {r}_{k}\end{eqnarray*}

(12) \begin{eqnarray*}{r}_{k}& = \frac{ \frac{1}{n-k} \ast \sum _{i=1}^{n-k} \left( {x}_{i}-\overline{x} \right) ({x}_{i+k}-\overline{x})}{ \frac{1}{n} \ast \sum _{i=1}^{n}{ \left( {x}_{i}-\overline{x} \right) }^{2}} \end{eqnarray*}

(13) \begin{eqnarray*}{Z}_{mk}& = \left\{ \begin{array}{@{}l@{}} \displaystyle \frac{{S}_{mk}-1}{\sqrt{Var \left( {S}_{mmk} \right) }} ;~~~~~if~{S}_{mk}\gt 0 \\ \displaystyle \\ \displaystyle 0;~~~~~if~{S}_{mk}=0 \\ \displaystyle \frac{{S}_{mk}+1}{\sqrt{Var \left( {S}_{mmk} \right) }} ;~~~~~if~{S}_{mk}\lt 0 \end{array} \right. \end{eqnarray*}

In the above formulas, n determines the amount of the data, and according to the test principle, the data values over time jth and kth, respectively, must be greater than 10, x_i_ and x_j_, and in $sgn \left( {x}_{j}-{x}_{i} \right) $, is the sgn function; as $Var \left( {S}_{mmk} \right) $, the variance value for modified MK, W reflects modified coefficient of auto-correlated data, *r*_*k*_ present the kth auto correlated coefficient, $\overline{x}$ Indicates mean of the series, P represents the number of bound groups, and ti represents the number of degrees one ties in the Mann-Kendall test evaluation rate; Similarly, equation 3’s positive value stipulates a growing drift, whereas a negative sign suggests a declining trend in the sequence. The standard normal distribution and the expected Z_cal_ value are compared according to the confidence limits (*α* =5% or *α* =1%) (Mann, 1945; Kendall, 1975; Helsel & Hirsch, 2002; Kampata, Parida & Moalafhi, 2008). If the calculated Z_cal_ value is more than —Z_MK_— < —Z_(1−*α*∕2)_—, and the null hypothesis (H_o_) is accepted.

#### Trend slope

The Sen slope is used to investigate the trend line, and its magnitude used in this research, based on research done by Theil (1950) and Sen (1968) calculated as described in the following equation:

(14) \begin{eqnarray*}{Q}_{\beta }=Median \left( \frac{{x}_{t}-{x}_{s}}{t-s} \right) for~all~s\lt t\end{eqnarray*}

Where, 1<s<t<n,

*Q*_*β*_ Illustrates the trend line estimator, and *x*_*t*,_Reflects the t^th^ data observed. The positive value of *Q*_*β*_ Demonstrates the upward trend direction and its magnitude, and a negative expresses a declining pattern (Yue, Pilon & Phinney, 2003).

## Results and Discussion

### Performance analysis for statistical probability distributions

In recent decades, reference evapotranspiration, drought, precipitation, and aridity index and its concern probability theory have seen renewed popularity (Blain, 2012; Blain & Meschiatti, 2014; Huang et al., 2017; Jiang et al., 2020; Nam & Choi, 2014; Rahman et al., 2013; Wu, Xiong & Wu, 2017; Zhang et al., 2015). Hence, it is vital to understand the role of regional ET probability distributions in both dry and wet conditions. This could provide a scientific foundation for the management and allocation of regional water resources. Thus, the analysis was carried out for the mean monthly ET_o_ values by FAO-56(PM), and Hargreaves-Samani (HS) estimates for twelve locations. A total of 120 months of the test data set has use for distribution adjustment. Tables 1 and 2 show the ET_o_ estimates’ descriptive statistics for the entire region’s study period. Further, the Tables S2 and S3 show the best fit data adjustment to each weather station’s probabilistic models based on the AD test (*p* < 0.05). According to the AD test, we have checked twenty-one probability distribution to tested stations and applied on a series of ET_o_ based on FAO-56(PM) and Hargreaves-Samani estimates. The top three fitted distributions out of twenty-one tested probability distributions were selected for every included station in the first task. For this purpose, we used the AD test in our analysis to determine FAO-56(PM) based ETo Probability distribution models, Hargreaves-Samani (HS) based ET_o_ Probability distribution models, and Seasonal established ET_o_ Probability distribution models for every location in the selected region. Table 2, results revealed that the Johnson SB distribution performed the best by occupying 50% of the total stations. However, the Generalized Pareto showed a 25% inclusion in the study region.

**Table 2:** Descriptive statistics during the entire study period.

**Stations**	**N**	**Var**	**Skewness**		**Kurtosis**	
Statistics		Statistic	Statistic	SE	Statistic	SE
Balakot	120	2.97	0.28	0.22	−0.66	0.44
Cherat	120	2.88	0.58	0.22	−0.35	0.44
Chitral	120	10.36	0.47	0.22	−0.93	0.44
DI Khan	120	3.95	0.41	0.22	−0.63	0.44
Dir	120	5.36	0.45	0.22	−0.64	0.44
Drosh	120	7.43	0.39	0.22	−1.01	0.44
Kakul	120	2.23	0.43	0.22	−0.58	0.44
Kohat	120	7.03	0.60	0.22	−0.28	0.44
Parachinar	120	4.12	0.72	0.23	−0.17	0.46
Peshawar	120	7.68	0.63	0.22	−0.65	0.44
Risalpur	120	56.08	3.84	0.23	17.28	0.46
Saidu Sharif	120	5.36	0.45	0.22	−0.64	0.44

In Contrast, we applied the same type of procedure for evapotranspiration estimates based on HS methodology and, the results have shown in the Table S2. The Generalized Pareto showed a 33% presence among all the stations. Besides, Generalized Gamma(4P) and Generalized Extreme Value contributed 16% in overall stations. Moreover, the probability distributions scheme has also been applied in distinct seasons such as Winter, Spring, Dry Summer, Monsoon, and Autumn. The results designate that the Cauchy distribution occupied 25%, Burr and Logistic 16% respectively among all the stations in the winter season. However, in the Spring season, the Burr contributed 33%, while Johnson SB and Generalized Extreme Value showed 16% of the total stations. During Pre-Monsoon, the Johnson SB and log Pearson-3 showed their presence at 41.6% and 25%, respectively. Apart from this, Johnson SB, Burr, and Cauchy performed 33%, 25%, and 16% in overall stations during the red summer. While in Post monsoon, the Burr showed 41.6%, Johnson SB, and Burr(4P) with 16% results among all the study regions. In the second stage, the same procedure was checked by the Kolmogorov–Smirnov(KS) test statistic with (*p* < 0.05) and the top three best-fitted distribution selected for every station. According to the KS test in the Tables S4 and S5, the results demonstrate that the Johnson SB distribution performed the best by occupying 58.33% of the whole study regions. Likewise, Generalized Pareto showed a 25% enclosure in the study area on top. Hence, Generalized Pareto with 50% performance makes it the second best-fit distribution in the entire study region.

In comparison, the HS ET_o_ estimates indicate that the Johnson SB occupies 91.6% presence among all the stations. The Generalized Pareto contributed 75% in overall stations holding the second position. However, based on seasonal ET_o_ estimates, Cauchy and Burr contributed 41.6% and 25% respectively during winter, while Generalized Extreme Value showed 41.6% and Gamma with 16% out of the overall study region in spring. In pre-monsoon, Generalized Extreme Value and Gumbel-Min showed their presence at 25% and 16%, respectively. While in red summer Generalized Extreme Value with 25% and Gumbel-Max showed its presence at 16%. Furthermore, the Burr and Generalized Extreme value distribution performed 41.6% and 16%, respectively, during post-monsoon.

In the present research analysis, according to the evidence provided by our goodness of fit tests, the top two Statistical probability distribution which offered the best adjustment to the more significant part of the study region based on mean monthly ET_o_ values were the Generalized Pareto and Johnson SB (4 parameters lognormal distribution). However, the Burr, Johnson SB, and GEV showed the best fit and great adjustment during the covered period. Therefore, the Johnson SB and Generalized Pareto took top place for the entire Khyber Pakhtunkhwa region. On the other hand, Burr, Johnson SB, and GEV have shown stunning results in different seasons in the whole sample space. For this reason, the scale parameters lambda and alpha (*λ*&*α*) and shape parameters Gamma and Beta (*γ* & *β*) for both the probability distribution function were obtained and evaluated by the Anderson Darling test at (*p* < 0.05). The parameter values are shown in Tables 3 and 4, for every weather station. In the meantime, It is exciting to note that the scale parameter lambda for Johnson SB contains the minimum and maximum values (7.62 & 12) with no significant variation except Risalpur. The scale parameter value approach (63.6), the highest one for the mean monthly ET_o_. However, the scale parameter (*α*) of Generalized Pareto distribution, shown no significant variation during the entire study period with a minimum value of 3.65 and maximum value of 6.88, respectively.

**Table 3:** Reference evapotranspiration (ETo) values by using Johnson SB distribution.

Stations	Johnson SB			
Parameters	*γ*(Shape)	*δ*(Shape)	*λ*(Scale)	*ξ*(location)
Balakot	0.4424	1.1088	9.1355	0.3493
Cherat	0.8248	1.0316	9.0851	0.8426
Chitral	0.4491	0.6301	12.000	0.1565
DI Khan	0.5666	0.9266	9.8679	0.2216
Dir	0.5786	0.9266	11.050	0.1107
Drosh	0.3734	0.6428	10.140	0.3610
Kakul	0.6109	1.0199	7.6258	0.1469
Kohat	0.8858	1.0706	14.726	0.0000
Parachinar	0.9326	0.9375	10.529	0.1121
Peshawar	0.6227	0.6751	11.107	0.7227
Risalpur	2.0715	0.4588	63.691	2.5855
Saidu Sharif	0.5766	0.9266	11.052	0.1107

**Table 4:** Reference evapotranspiration (ETo) values by using Generalized Pareto distribution.

Stations	Generalized Pareto		
Parameters	Shape (*β*)	Scale (*α*)	location (*μ*)
Balakot	0.7865	4.8854	0.7149
Cherat	0.5154	3.6518	1.5088
Chitral	0.5028	6.8853	0.1282
DI Khan	0.6548	4.9774	1.0011
Dir	0.6135	5.5423	0.8345
Drosh	0.5913	6.4104	0.3937
Kakul	0.6530	3.7186	0.7776
Kohat	0.5313	5.8166	1.0864
Parachinar	0.4186	3.9136	0.5931
Peshawar	0.4067	5.2887	0.7537
Risalpur	0.2863	3.7505	0.9676
Saidu Sharif	0.6135	5.5423	0.8345

**Figure 2:**
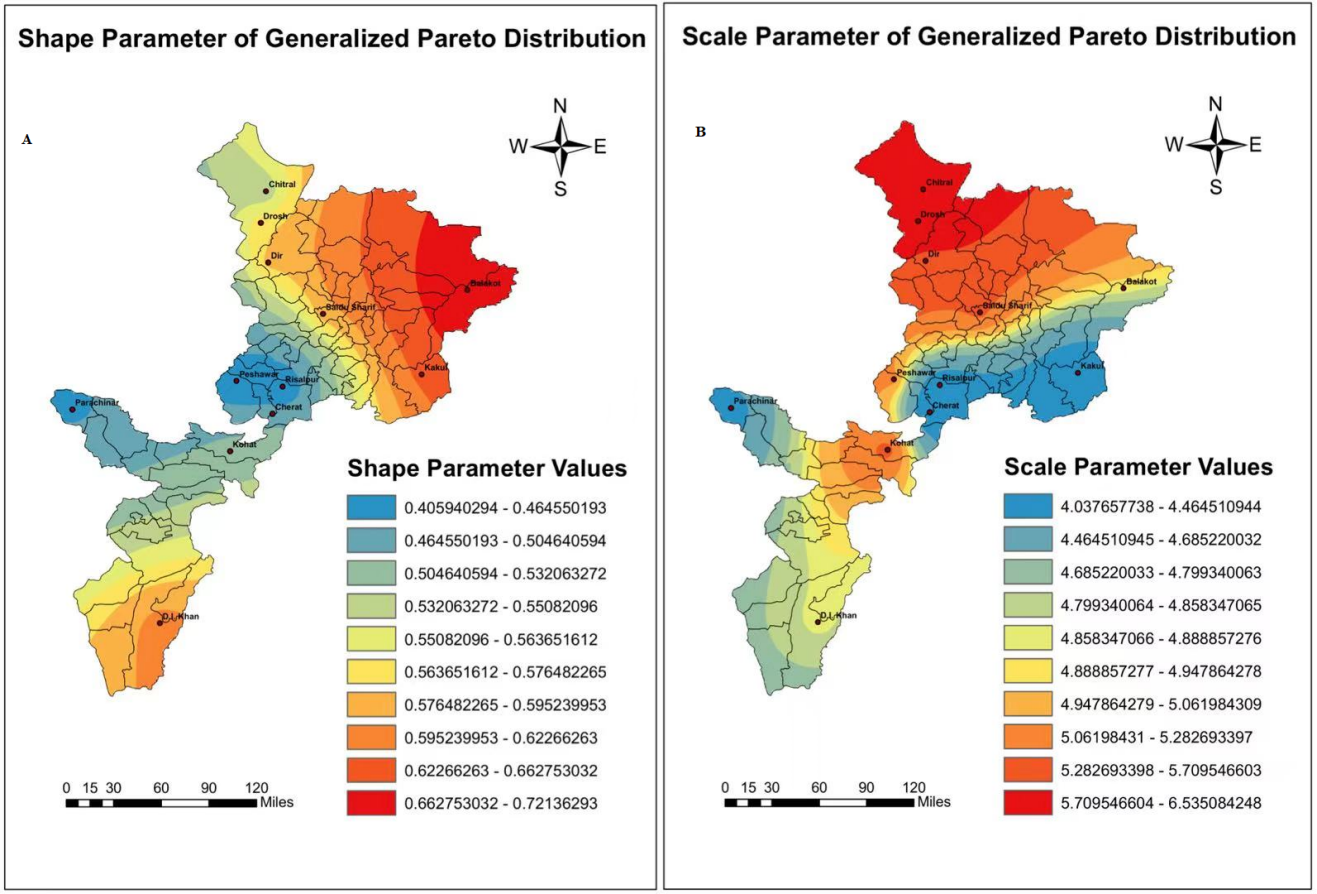
Bayesian Kriging-based spatial interpolation maps of scale and shape parameters values in millimeters of Generalized Pareto probability distribution. Simultaneously, the legends showed changes in different parameter values regarding reference evapotranspi.

**Figure 3:**
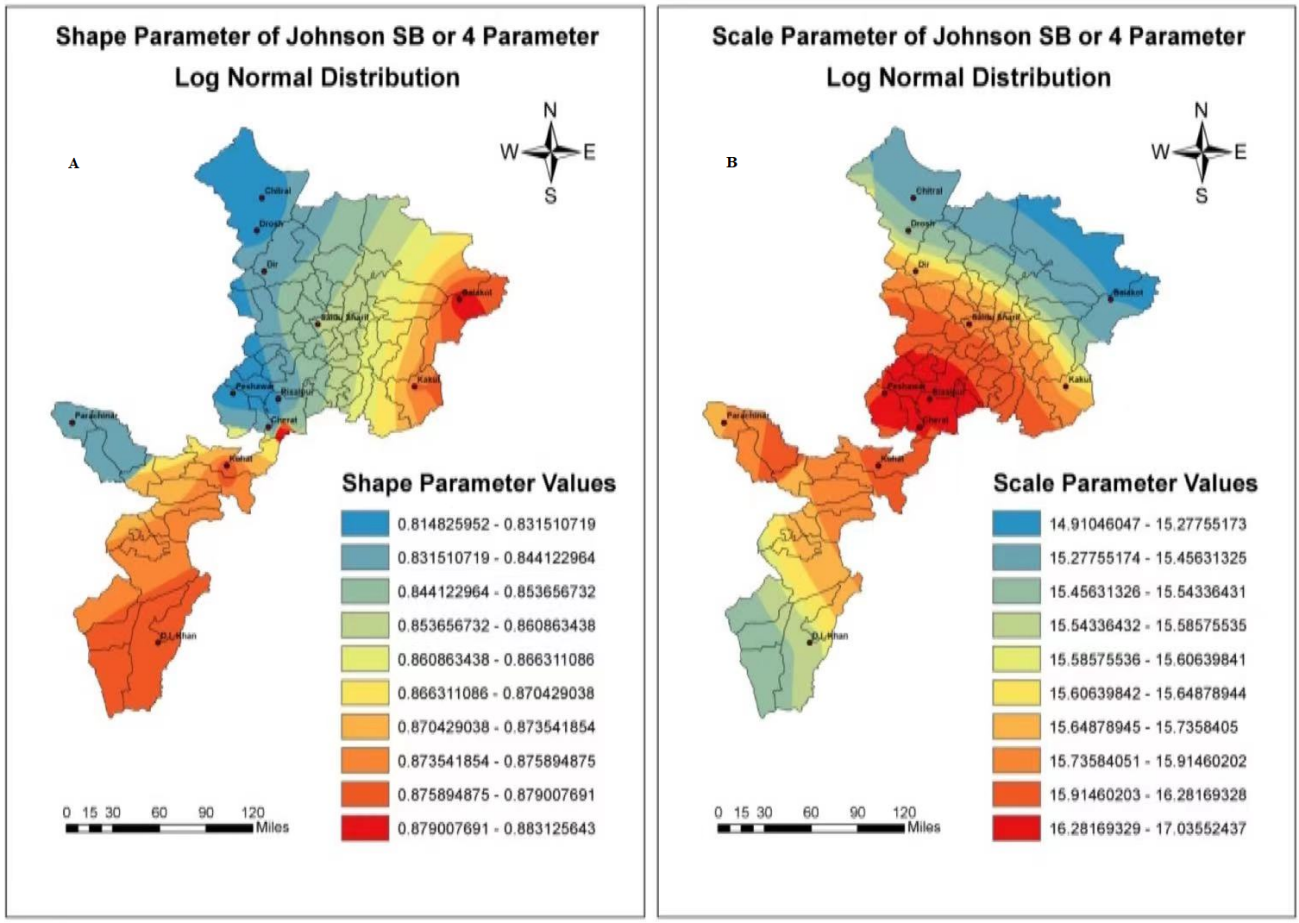
Bayesian Kriging-based spatial interpolation maps of scale and shape parameters values in millimeters of Johnson SB probability distribution. Simultaneously, the legends showed different parameter values regarding reference evapotranspiration changes for.

Besides, the shape parameter Beta (*β*) of Generalized Pareto showed no significant variation. The minimum-maximum values lie between (0.2863 & 0.7865) for the mean monthly ET_o_ series during the study period. Further, the shape parameter of Johnson SB also showed slice difference by containing minimum and maximum values (0.3734 & 2.0715), respectively. In conclusion, the spatial distribution maps regarding the scale parameters (*λ*& *α*) and shape parameters (*γ* & *β*) of Generalized Pareto & Johnson SB shown in Figs. 2 and 3.

### Reference evapotranspiration trends analysis

Pakistan has ranked 18th among the most susceptible countries in the Global Climate Risk Index in 2011. The state is on the third highly critical threat in 180 nations, impacted by weather losses (Kreft, Eckstein & Melchior, 2013). Consequently, it is essential to consider ET_o_ trends and their implications for territorial dry and wet circumstances. This could provide an objective validity for regional water resource management and allocation, as well as for scientific judgment linked with flood and drought disaster prevention (Ma et al., 2017). This research aims to conduct a temporal trend analysis of reference evapotranspiration in Khyber Pakhtunkhwa using the FAO-56 Penman-Monteith equation to assess their trends over the study period. Therefore, we applied the Mann-Kendall trend test and the Sen slope estimator to find more information about the regional reference evapotranspiration trends. The MK and Sen’s slope estimator (Q_β_) are determined using the mean monthly evapotranspiration results of ten years. Table 5 summarizes the findings for 12 stations in this respect. This list demonstrates two-tailed confidence patterns (5%) for the overall data series in all the locations. According to the Mann Kendall test at a 5% level of significance, the entire study region showed insignificant upward and downward mean monthly evapotranspiration trends in the concerned period. To accurately identify the trend’s magnitude and direction, we used the Sen Slope estimator. Based on the Sen slope estimator, five study locations, including Drosh, Kakul, Kohat, Parachinar, and Risalpur, showed an increasing trend in the overall testing period.

**Table 5:** Mann-Kendall and Sen Slope estimator outputs for the study location.

Stations\Test	Kendall’s tau	*P*-value	Sen’s slope	LCL	UCL	H_o_/ H_a_
Balakot	−0.050	0.674	−0.025	−0.045	0.162	H_o_ Accepted
Cherat	−0.024	0.781	−0.015	−0.035	0.183	H_o_ Accepted
Chitral	−0.057	0.750	−0.068	−0.106	0.252	H_o_ Accepted
Di khan	−0.053	0.707	−0.032	−0.056	0.182	H_o_ Accepted
Dir	−0.007	0.903	−0.003	−0.028	0.246	H_o_ Accepted
Drosh	0.075	0.711	0.075	0.041	0.410	H_o_ Accepted
Kakul	0.001	0.991	0.005	−0.015	0.176	H_o_ Accepted
Kohat	0.017	0.888	0.033	−0.005	0.345	H_o_ Accepted
Parachinar	−0.004	0.951	0.001	−0.029	0.276	H_o_ Accepted
Peshawar	−0.024	0.689	−0.012	−0.035	0.277	H_o_ Accepted
Risalpur	0.002	0.980	0.012	−0.020	0.312	H_o_ Accepted
Saidu sharif	−0.017	0.806	−0.010	−0.037	0.238	H_o_ Accepted

**Notes.**

LCL and UCL means lower and upper control limits, H_o_: used for the null hypothesis, and H_*a*_ used for the alternative hypothesis based on a 5% level of significance.

**Figure 4:**
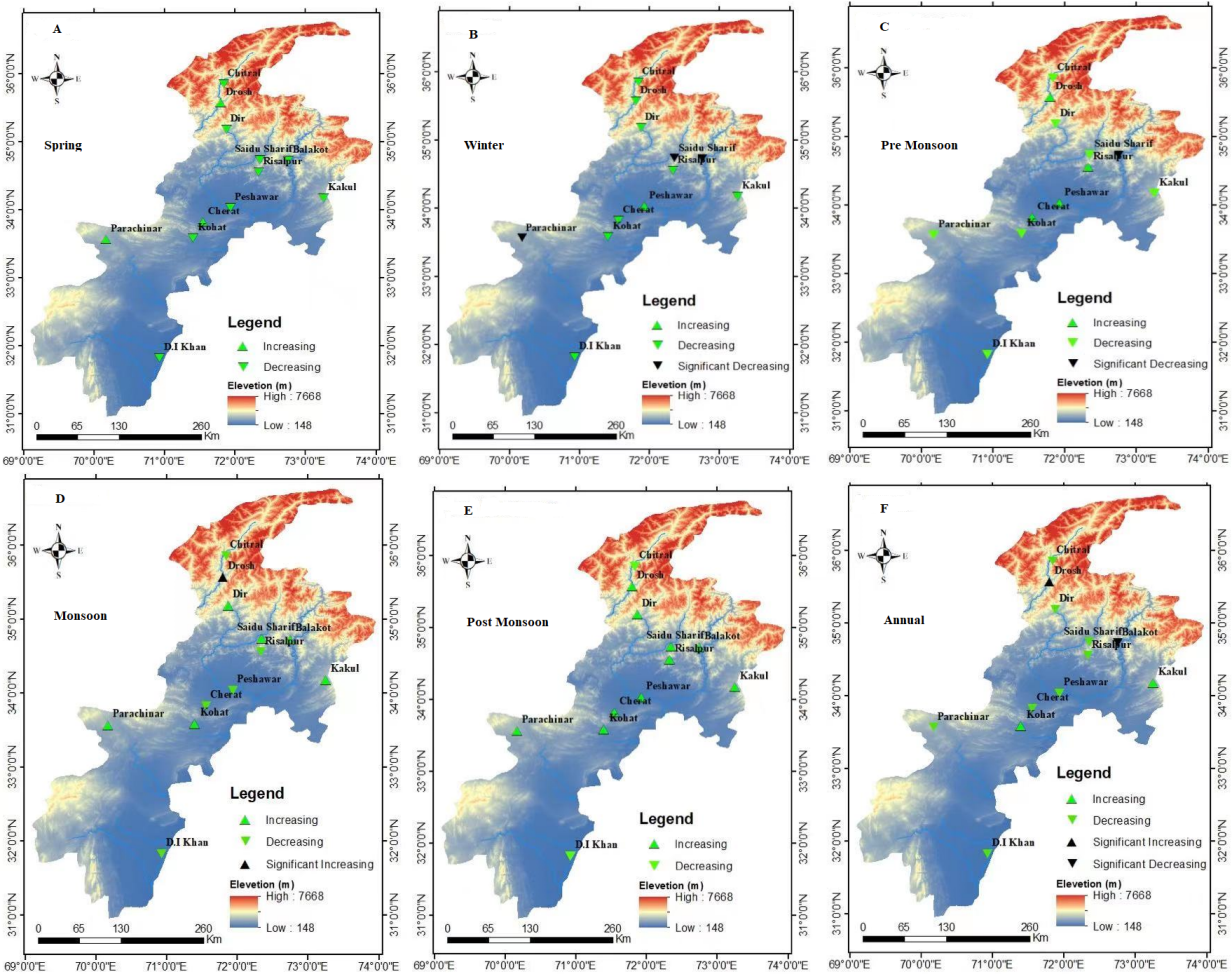
Seasonal distribution of *ET*_*o*_ trends in the study area. The black triangle symbol shows the statistically significant increasing or decreasing trends, and the green triangle indicates the insignificant trends (A–F).

In Contrast, seven study regions, such as Balakot, Cherat, Chitral, DI Khan, Dir, Peshawar, and Saidu Sharif, showed a decreasing trend. In conclusion, the Seasonal distribution of ET_o_ trends in the study area is illustrated graphically in Fig. 4. The black triangle symbol shows the statistically significant increasing or decreasing trends, and the green triangle indicates the insignificant trends. The overall monthly, seasonal, and annual trends are discussed one by one in the following sub-sections.

#### Monthly evapotranspiration trend analysis

The evaluation of trend was carried out each month at every station; the results showed in Tables 6, 7, 8 and 9. According to the output, in January, 8 out of 12 (66.6%) locations showed insignificant negative trends, while Kohat, DI Khan, Risalpur, and Peshawar indicated positive drifts with an overall 33.3%. Similarly, in February, overall, 9 (75%) stations revealed a negative trend, and three (25%) showed a positive direction. Further, March’s month showed 8 (66.6%) out of 12 locations showed insignificant negative trends than Cherat. Drosh, Parachinar, and Peshawar indicated positive behavior of movement, while in April total of 10 (83.3%) study regions out of 12 showed an overall negative trend. Likewise, both showed an overall (50%, 33.3%) negative and (50%, 66.6%) positive trend behavior in the entire study area in May and June. Drosh showed positive statistically significant trends at a 5% level of significance with Kendall tau (0.68, 0.78, and 0.78) and Sen slope values (0.31, 0.38, and 0.29) mm over July, August, and September, respectively. Overall (16.6%, 66.6%, and 33.3%) and (83.3%, 33.3%, and 66.6%) downward and upward drifts were observed in the same months, respectively. Moreover, a total (83.3%, 41.6%, and 25%) stations showed positive and (16.6%, 58.3%, and 75%) revealed negative trends, respectively, over October, November, and December.

**Table 6:** Monthly wise results for Mann-Kendall and Sen Slope estimator.

Stations	Tests	January	February	March	April	May	June
	Kendall’s tau	−0.378	−0.467	−0.200	−0.333	−0.467	−0.111
Balakot	*p*-value	0.152	0.074	0.474	0.210	0.074	0.721
	Sen’s slope	−0.020	−0.036	−0.075	−0.128	−0.239	−0.064
	Kendall’s tau	−0.244	−0.022	0.111	−0.067	0.156	0.111
Cherat	*p*-value	0.371	1.000	0.721	0.858	0.102	0.721
	Sen’s slope	−0.057	−0.014	0.040	−0.025	0.057	0.045
	Kendall’s tau	−0.511	−0.422	−0.111	−0.289	−0.333	−0.467
Chitral	*p*-value	0.049	0.107	0.721	0.283	0.210	0.074
	Sen’s slope	−0.065	−0.057	−0.077	−0.106	−0.396	−0.346
	Kendall’s tau	0.200	−0.111	−0.244	−0.289	−0.333	−0.022
DI Khan	*p*-value	0.474	0.186	0.371	0.283	0.210	1.000
	Sen’s slope	0.024	−0.035	−0.067	−0.111	−0.189	−0.023
	Kendall’s tau	−0.378	−0.156	−0.067	−0.200	−0.244	0.022
Dir	*p*-value	0.152	0.592	0.858	0.474	0.371	1.000
	Sen’s slope	−0.042	−0.011	−0.032	−0.072	−0.103	0.010
	Kendall’s tau	−0.289	−0.111	0.067	0.111	0.022	0.200
Drosh	*p*-value	0.283	0.721	0.858	0.721	1.000	0.474
	Sen’s slope	−0.054	−0.011	0.003	0.048	0.042	0.071

**Table 7:** Monthly wise results for Mann-Kendall and Sen Slope estimator.

Stations	Tests	July	August	September	October	November	December
	Kendall’s tau	0.200	0.333	0.244	0.156	0.022	−0.422
Balakot	*p*-value	0.474	0.210	0.371	0.107	1.000	0.107
	Sen’s slope	0.018	0.013	0.042	0.047	0.000	−0.036
	Kendall’s tau	−0.067	−0.067	−0.378	0.111	−0.378	0.022
Cherat	*p*-value	0.858	0.858	0.152	0.721	0.152	1.000
	Sen’s slope	−0.034	−0.026	−0.102	0.015	−0.109	0.003
	Kendall’s tau	0.022	−0.156	−0.289	−0.244	0.067	−0.289
Chitral	*p*-value	1.000	0.393	0.283	0.371	0.858	0.283
	Sen’s slope	0.006	−0.094	−0.138	−0.049	0.008	−0.018
	Kendall’s tau	−0.067	−0.289	−0.067	−0.022	−0.156	−0.422
DI Khan	*p*-value	0.858	0.283	0.858	1.000	0.592	0.107
	Sen’s slope	−0.014	−0.029	−0.007	−0.004	−0.030	−0.027
	Kendall’s tau	0.067	−0.156	0.244	0.111	−0.111	0.111
Dir	*p*-value	0.858	0.427	0.371	0.721	0.721	0.721
	Sen’s slope	0.009	−0.005	0.068	0.077	−0.020	0.019
	Kendall’s tau	0.689	0.778	0.689	0.422	−0.111	0.156
Drosh	*p*-value	**0.007**^∗^	**0.002**^∗^	**0.007**^∗^	0.107	0.721	0.592
	Sen’s slope	0.310	0.380	0.29	0.200	−0.020	0.025

**Table 8:** Monthly wise results for Mann-Kendall and Sen Slope estimator.

Stations	Tests	January	February	March	April	May	June
	Kendall’s tau	−0.111	0.022	−0.067	−0.111	0.022	0.156
Kakul	*p*-value	0.721	1.000	0.858	0.721	1.000	0.592
	Sen’s slope	−0.017	0.000	−0.022	−0.020	0.003	0.046
	Kendall’s tau	0.111	0.022	−0.067	−0.244	−0.022	0.289
Kohat	*p*-value	0.721	1.000	0.858	0.371	1.000	0.283
	Sen’s slope	0.015	0.02	−0.053	−0.121	−0.037	0.241
	Kendall’s tau	−0.467	−0.289	0.200	0.022	0.022	−0.067
Parachinar	*p*-value	0.074	0.283	0.474	1.000	1.000	0.858
	Sen’s slope	−0.110	−0.041	0.053	0.006	0.039	−0.054
	Kendall’s tau	0.244	0.067	0.022	−0.289	−0.022	0.511
Peshawar	*p*-value	0.371	0.858	1.000	0.283	1.000	0.049
	Sen’s slope	0.035	0.068	0.004	−0.150	0.000	0.270
	Kendall’s tau	0.022	−0.067	−0.111	−0.022	0.156	0.244
Risalpur	*p*-value	1.000	0.858	0.721	1.000	0.592	0.371
	Sen’s slope	0.003	−0.016	−0.024	−0.018	0.085	0.176
	Kendall’s tau	−0.378	−0.156	−0.067	−0.200	−0.244	0.022
Saidu Sharif	*p*-value	0.152	0.592	0.858	0.474	0.371	1.000
	Sen’s slope	−0.042	−0.011	−0.032	−0.072	−0.103	0.010

**Table 9:** Monthly wise results for Mann-Kendall and Sen Slope estimator.

Stations	Tests	July	August	September	October	November	December
	Kendall’s tau	0.156	0.200	0.200	0.111	0.200	−0.022
Kakul	*p*-value	0.592	0.474	0.474	0.721	0.474	1.000
	Sen’s slope	0.008	0.008	0.040	0.028	0.026	−0.001
	Kendall’s tau	0.111	0.156	0.422	0.333	−0.111	−0.378
Kohat	*p*-value	0.721	0.592	0.107	0.210	0.721	0.152
	Sen’s slope	0.069	0.040	0.096	0.127	−0.021	−0.059
	Kendall’s tau	0.200	−0.111	0.156	0.200	0.156	−0.511
Parachinar	*p*-value	0.474	0.721	0.592	0.474	0.592	0.049
	Sen’s slope	0.142	−0.055	0.048	0.070	0.033	−0.161
	Kendall’s tau	0.156	−0.289	0.200	0.156	−0.022	−0.333
Peshawar	*p*-value	0.592	0.283	0.474	0.592	1.000	0.210
	Sen’s slope	0.031	−0.106	0.026	0.070	−0.021	−0.031
	Kendall’s tau	0.067	−0.067	−0.022	0.244	0.067	−0.111
Risalpur	*p*-value	0.858	0.858	1.000	0.371	0.858	0.721
	Sen’s slope	0.052	−0.029	−0.003	0.093	0.004	−0.022
	Kendall’s tau	0.067	−0.156	0.244	0.111	−0.111	−0.067
SaiduSharif	*p*-value	0.858	0.427	0.371	0.721	0.721	0.858
	Sen’s slope	0.009	−0.005	0.068	0.077	−0.020	−0.011

#### Seasonal and annual evapotranspiration trends analysis

The seasons in Pakistan include winter (December to February), Spring (March to April), Pre-monsoon (Dry Summer) period (May to June), Monsoon period (July to September), and Post-Monsoon (autumn) from (October to November). The seasonal evapotranspiration trends in the Northwest region of Pakistan have checked using the same framework (MK and Q_*β*_) shown in Tables 10 and 11. The statistically significant and insignificant trends are graphically presented in Fig. 4, respectively. The reference evapotranspiration showed negative patterns over 91.6 percent of stations overall, based on Mann Kendall and Sen slope seasonal analysis during the winter season. Only 8.33 percent of stations showed a positive trend. Balakot, Parachinar, and Saidu Sharif showed statistically significant negative trends results with Sen slope values (−0.068 mm, −0.334 mm, and −0.067 mm) per season at a 5% significance level. Overall an insignificant negative trend in evapotranspiration was observed in 75% of stations, while 25% showed positive behavior during the Spring season.

**Table 10:** Seasonal ET trend analysis by using Mann-Kendall and Sen Slope approach.

Stations	Tests	Winter	Spring	Dry summer	Monsoon	Autumn	Annual
	Kendall’s tau	−0.6	−0.422	−0.6	0.067	0.111	−0.511
Balakot	*p*-value	0.02^*^	0.107	0.02^*^	0.858	1	0.049^*^
	Sen’s slope	−0.068	−0.154	−0.457	0.037	0.052	−0.508
	Kendall’s tau	−0.111	0.022	0.022	−0.111	0.067	−0.022
Cherat	*p*-value	0.721	1	1	0.721	0.858	1
	Sen’s slope	−0.093	0.009	0.084	−0.117	0.045	−0.08
	Kendall’s tau	−0.333	−0.289	−0.333	−0.289	−0.156	−0.289
Chitral	*p*-value	0.21	0.283	0.21	0.283	0.592	0.283
	Sen’s slope	−0.153	−0.191	−0.577	−0.279	−0.049	−1.221
	Kendall’s tau	−0.289	−0.378	−0.244	−0.156	−0.111	−0.289
DI Khan	*p*-value	0.283	0.152	0.371	0.592	0.721	0.283
	Sen’s slope	−0.037	−0.227	−0.229	−0.072	−0.029	−0.453
	Kendall’s tau	−0.244	−0.244	−0.2	0.111	0.156	−0.022
Dir	*p*-value	0.371	0.371	0.474	0.721	1	1
	Sen’s slope	−0.014	−0.06	−0.133	0.038	0.025	−0.022
	Kendall’s tau	−0.156	0.111	0.067	0.822	0.244	0.733
Drosh	*p*-value	0.592	0.721	0.858	0.001^*^	0.371	0.004^*^
	Sen’s slope	−0.014	0.027	0.083	0.944	0.246	0.95

**Notes.**

* Significant trend at 5% level of significance.

**Table 11:** Seasonal ET trend analysis by using Mann-Kendall and Sen Slope approach.

Stations	Tests	Winter	Spring	Dry Summer	Monsoon	Autumn	Annual
	Kendall’s tau	−0.022	−0.022	−0.067	0.156	0.200	0.067
Kakul	*p*-value	1.000	1.000	0.858	0.592	0.474	0.858
	Sen’s slope	−0.004	−0.03	−0.032	0.035	0.052	0.193
	Kendall’s tau	−0.067	−0.111	−0.022	0.156	0.333	0.067
Kohat	*p*-value	0.858	0.605	1.000	0.592	0.210	0.858
	Sen’s slope	−0.017	−0.164	−0.055	0.255	0.168	0.481
	Kendall’s tau	−0.689	0.200	−0.067	0.111	0.200	−0.022
Parachinar	*p*-value	0.007^*^	0.474	0.858	0.721	0.474	1.000
	Sen’s slope	−0.334	0.055	−0.241	0.061	0.069	−0.110
	Kendall’s tau	0.200	−0.156	0.200	−0.244	0.067	−0.111
Peshawar	*p*-value	0.474	1.000	0.474	0.371	0.858	0.288
	Sen’s slope	0.093	−0.051	0.199	−0.230	0.030	−0.072
	Kendall’s tau	−0.111	−0.022	0.200	−0.111	0.244	−0.022
Risalpur	*p*-value	0.721	1.000	0.474	0.688	0.371	1.000
	Sen’s slope	−0.094	−0.004	0.255	−0.080	0.101	−0.035
	Kendall’s tau	−0.644	−0.244	−0.200	0.111	0.156	−0.111
Saidu Sharif	*p*-value	0.012^*^	0.371	0.474	0.721	1.000	0.721
	Sen’s slope	−0.067	−0.060	−0.133	0.038	0.025	−0.169

**Notes.**

* Significant trend at 5% level of significance.

**Figure 5:**
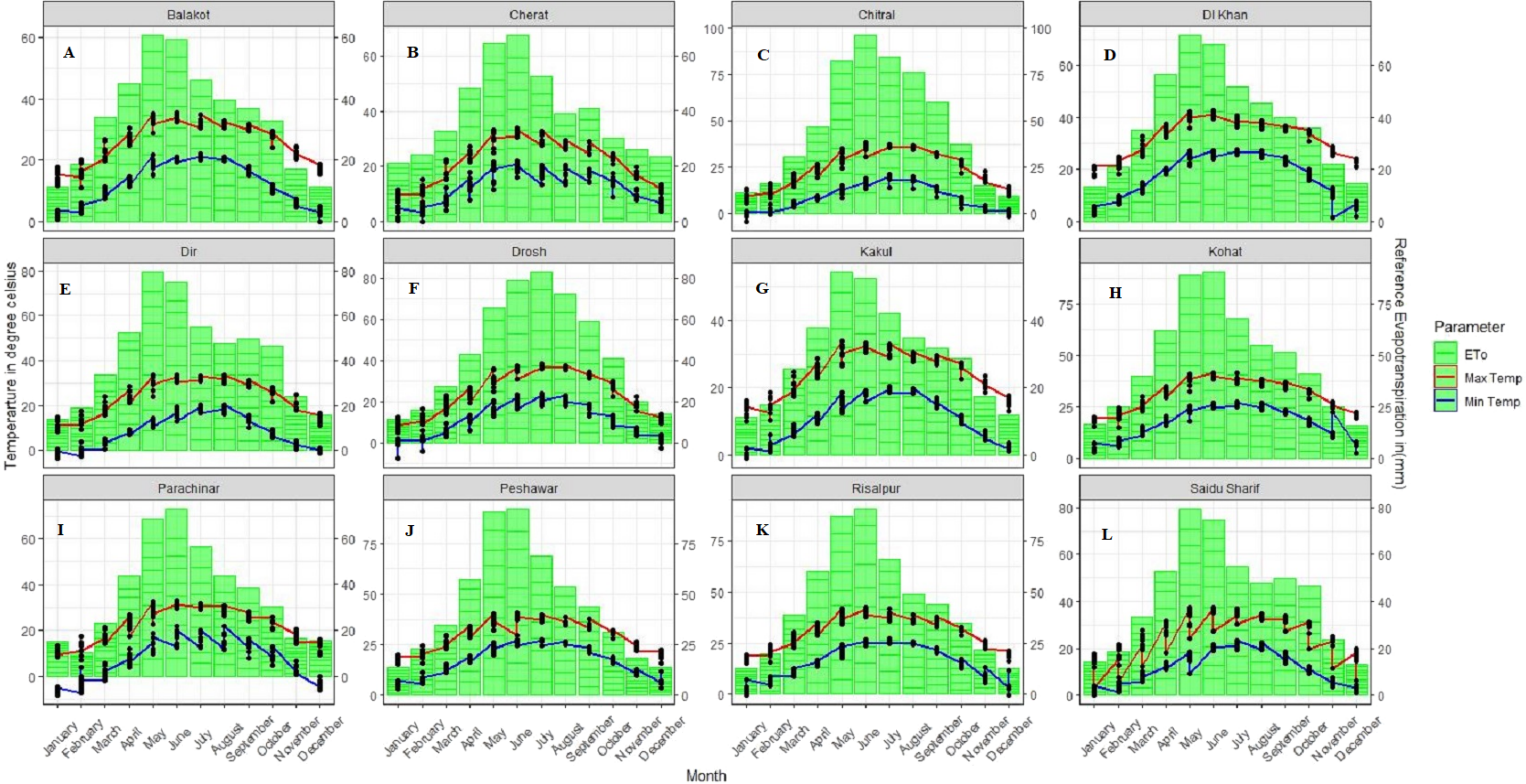
Climograph of temperature and reference evapotranspiration. The blue color represents minimum temperature, the red color represents maximum temperature, while the bars stand for reference evapotranspiration. It is important to note that this is the minimu (A–L).

Further, Balakot showed a negative statistically significant trend with Sen Slope (−0.457 mm) in the Dry Summer, while 66% of stations revealed statistically insignificant trends in the same time season. In Monsoon, Drosh showed a positive statistical significant trend with Sen slope value (0.944 mm), while 58.3% of stations showed insignificant positive trends during the study period. However, almost 83.33% of stations revealed positive statistically insignificant trends; the rest showed negative autumn trends. Comparatively, the Annual evapotranspiration trends showed negative behavior over 75% of stations. Balakot showed a statistically significant negative trend with Sen slope value (−0.508 mm) over an annual period. In contrast, a statistically significant positive trend was present in the Drosh region with a Sen slope value (0.95 mm). Finally, a climatic analysis of temperature and reference evapotranspiration for the whole study region is shown in Fig. 5.

## Summary and Conclusion

This paper has provided a search of the best fit distribution for the reference evapotranspiration and a thorough analysis of twelve meteorological stations of Khyber Pakhtunkhwa. We applied the twenty-one statistical probability distribution at each station and selected the most appropriate according to KS and AD fit tests approach. We also checked the ET trends through Mann-Kendall and Sen slope trend analysis. In general, we found that Johnson SB and Generalized Pareto probability distributions’ performance is a better fit and adjusted to the mean monthly ETo. In seasonal ETo estimates, the Burr, Johnson SB, and GEV showed the best results.

Besides, compared to the monthly time series, the seasonal and annual time series appear to have a more significant trend variation. At 91.66% of stations, the winter season tended to decrease, while mixtures of rising and falling trends has observed in spring and autumn. Also, 83.3 percent of stations in the Post Monsoon season indicated rising drifts. The analysis found a reducing trend pattern identified on average in winter, spring, and annual cycles. The preliminary findings demonstrated that 11 out of 12 stations on average exhibited a downward trend in ET, with three statistically significant tests observed in winter and Monsoon at Balakot, Drosh, Parachinar, and Saidusharif. Analogously, the high rate of positive patterns in the warm seasons could lead to good crops and greenery in the whole area with sufficient rain. Thus, the high return of the flow distribution would be appropriate.

The province’s agriculture and economy rely predominantly on rainfall as a significant water source; any deficit, reduction, and decrease in ET_o_ or decrease in precipitation may contribute to water supplies scarcity, desertification, intermittent drought, and damaged savannas. It also ensures that agriculture will need more natural water consumption, which is already constrained. Furthermore, as a recommendation, a reasonable adaptive capacity such as irrigation maintenance, soil water ecological restoration, reliable agricultural production, change in plant species, sufficient crop intensities would be required to cope with the potential impacts. Further research must commence for assessing the proposed scheme to other locations in the country. In summing up, these research findings might be helpful in water resource management, water engineering, and policymakers for future planning.

##  Supplemental Information

10.7717/peerj.11597/supp-1 Supplemental Information 1 Overall geographical description about selected stations

10.7717/peerj.11597/supp-2 Supplemental Information 2 Top three statistical probability distributions based on Anderson Darling goodness of fit test by using FAO-56(PM) and Hargreaves-Samani estimation method

10.7717/peerj.11597/supp-3 Supplemental Information 3 Top three statistical probability distributions based on Kolmogorove Smirnov goodness of fit test by using FAO-56(PM) and Hargreaves-Samani estimation method

10.7717/peerj.11597/supp-4 Supplemental Information 4 Seasonal Reference Evapotranspiration (ETo) Probability Distribution by using Anderson Darling goodness of fit Statistic for statistical probability distribution selection

10.7717/peerj.11597/supp-5 Supplemental Information 5 Seasonal Reference Evapotranspiration (ETo) Probability Distribution by using Kolmogorove Smirnov goodness of fit Statistic for statistical probability distribution selection

## Additional Information and Declarations

### Data Availability

The following information was supplied regarding data availability: We used a ten year dataset of wind speed, air temperature, rainfall and relative humidity from 2000-2009 for ET estimation. The weather stations accessed include: Balakot, Cherat, Chitral, DI Khan, Dir, Drosh, Kakul, Kohat, Parachinar, Peshawar, Risalpur, and Saidu Sharif in the system. The general data for current months are available at the Pakistan Meteorological Department (http://www.pmd.gov.pk): 1. http://www.ffd.pmd.gov.pk/ffd_temps/temps_Apr.htm
2. http://www.ffd.pmd.gov.pk/ffd_rainfall/rainfall_Apr.htm
3. http://rmcpunjab.pmd.gov.pk/rmclhr/asppages/selectsynop.asp
4. http://nwfc.pmd.gov.pk/new/daily-forecast.php
5. https://nwfc.pmd.gov.pk/new/monthly-reports.php.
