# Supplementary material for: Design and analysis of statistical probability distribution and non-parametric trend analysis for reference evapotranspiration
Source: PeerJ. 2021 Jun 18;9:e11597. doi: 10.7717/peerj.11597 (PMC8216168; doi:10.7717/peerj.11597)
Supplement: Supplemental Information 1 [file peerj-09-11597-s001.docx]

| Station | Northern Latitude | Eastern Longitude | Elevation (m) |
| --- | --- | --- | --- |
| Balakot | 34^o^33^'^ | 72^o^20^'^ | 995 |
| Cherat | 33^o^49^'^ | 71^o^33^'^ | 1372 |
| Chitral | 35^o^51^'^ | 71^o^50^'^ | 1500 |
| D.I Khan | 31^o^49^'^ | 70^o^55^'^ | 173 |
| Dir | 35^o^11^'^ | 71^o^52^'^ | 1375 |
| Drosh | 35^o^34^'^ | 71^o^48^'^ | 1465 |
| Kakul | 34^o^10^'^ | 73^o^15^'^ | 1309 |
| Parachinar | 33^o^52^'^ | 70^o^40^'^ | 1725 |
| Peshawar | 34^o^02^'^ | 71^o^56^'^ | 327 |
| Risalpur | 34^o^04^'^ | 71^o^99^'^ | 317 |
| Saidu Sharif | 34^o^44^'^ | 72^o^21^'^ | 961 |
| Kohat | 33 ^o^35^'^ | 71 ^o^29^'^ | 508 |

**Note: Geographical information are taken from http://www.pmd.gov.pk/en/**
