# Supplementary material for: Design and analysis of statistical probability distribution and non-parametric trend analysis for reference evapotranspiration
Source: PeerJ. 2021 Jun 18;9:e11597. doi: 10.7717/peerj.11597 (PMC8216168; doi:10.7717/peerj.11597)
Supplement: Supplemental Information 2 [file peerj-09-11597-s002.docx]

| **Stations** | **PM based ETo** | **HS based ETo** |
| --- | --- | --- |
| Balakot | Johnson SB | Generalized Extreme Value |
|  | Normal | Normal |
|  | Rayleigh | Logistic |
| Cherat | Gamma | Generalized Pareto |
|  | Generalized Extreme Value | Weibull |
|  | Gamma(3p) | Generalized Extreme Value |
| Chitral | Generalized Pareto | Generalized Pareto |
|  | Weibull | Weibull |
|  | Burr | Generalized Extreme Value |
| DI Khan | Johnson SB | Weibull |
|  | Rayleigh | Generalized Extreme Value |
|  | Weibull | Normal |
| Dir | Generalized Pareto | Generalized Pareto |
|  | Johnson SB | G.Gamma(4p) |
|  | Weibull | Rayleigh |
| Drosh | Weibull | Generalized Pareto |
|  | Gamma(3p) | Weibull |
|  | Burr(4p | Generalized Extreme Value |
| Kakul | Johnson SB | Johnson SB |
|  | Rayleigh | Generalized Pareto |
|  | Burr | Weibull |
| Parachinar | Johnson SB | Rayleigh |
|  | Burr(4p) | Weibull |
|  | Weibull(3p) | Generalized Extreme Value |
| Peshawar | Johnson SB | G.Gamma(4p) |
|  | Generalized Pareto | Weibull |
|  | Burr(4p) | Generalized Extreme Value |
| Risalpur | Log Normal | Johnson SB |
|  | Generalized Extreme Value | Generalized Extreme Value |
|  | Burr | Normal |
| Saidu Sharif | Generalized Pareto | G.Gamma(4p) |
|  | Johnson SB | Weibull |
|  | Weibull | Log Pearson-3 |
| Kohat | Johnson SB | Generalized Extreme Value |
|  | Burr | Normal |
|  | Rayleigh | Logistic |

**Note:** Here 2p, 3p or 4p means two, three or four parameters.
