# Supplementary material for: Design and analysis of statistical probability distribution and non-parametric trend analysis for reference evapotranspiration
Source: PeerJ. 2021 Jun 18;9:e11597. doi: 10.7717/peerj.11597 (PMC8216168; doi:10.7717/peerj.11597)
Supplement: Supplemental Information 3 [file peerj-09-11597-s003.docx]

| **Stations** | **PM based ETo** | **HS based ETo** |
| --- | --- | --- |
| Balakot | Generalized Pareto | Johnson SB |
|  | Johnson SB | Generalized Pareto |
|  | Rayleigh | Weibull |
| Cherat | Johnson SB | Johnson SB |
|  | Generalized Pareto | G.Gamma(4p) |
|  | Log Normal | Generalized Pareto |
| Chitral | Johnson SB | Johnson SB |
|  | Generalized Pareto | Generalized Pareto |
|  | Weibull | Weibull |
| DI Khan | Weibull | Johnson SB |
|  | Johnson SB | Generalized Pareto |
|  | Generalized Pareto | G.Gamma(4p) |
| Dir | Generalized Pareto | Johnson SB |
|  | Johnson SB | Generalized Pareto |
|  | Burr | G.Gamma(4p) |
| Drosh | Johnson SB | Johnson SB |
|  | Generalized Pareto | Generalized Pareto |
|  | Weibull | Weibull |
| Kakul | Johnson SB | Johnson SB |
|  | Rayleigh | Generalized Pareto |
|  | Generalized Pareto | Rayleigh |
| Parachinar | Generalized Pareto | Johnson SB |
|  | Johnson SB | Generalized Pareto |
|  | Gamma(3p) | Rayleigh |
| Peshawar | Johnson SB | Johnson SB |
|  | Generalized Pareto | Generalized Pareto |
|  | Log pearson-3 | G.Gamma(4p) |
| Risalpur | Log Normal | Johnson SB |
|  | Generalized Extreme Value | Generalized Extreme Value |
|  | Log pearson-3 | Rayleigh |
| Saidu Sharif | Johnson SB | Johnson SB |
|  | Generalized Pareto | Generalized Pareto |
|  | Burr | G.Gamma(4p) |
| Kohat | Johnson SB | Generalized Pareto |
|  | Generalized Pareto | Johnson SB |
|  | Weibull | Rayleigh |
