# Supplementary material for: Design and analysis of statistical probability distribution and non-parametric trend analysis for reference evapotranspiration
Source: PeerJ. 2021 Jun 18;9:e11597. doi: 10.7717/peerj.11597 (PMC8216168; doi:10.7717/peerj.11597)
Supplement: Supplemental Information 4 [file peerj-09-11597-s004.docx]

| **Stations** | **Winter** | **Spring** | **Dry Summer** | **Monsoon** | **Autumn** |
| --- | --- | --- | --- | --- | --- |
| **Balakot** | Burr(4P) | Burr | LP-3 | Johnson SB | Burr |
| **Cherat** | Burr(4P) | Johnson SB | LP-3 | Normal | Burr(4P) |
| **Chitral** | G. Pareto | Johnson SB | Johnson SB | Johnson SB | Johnson SB |
| **DI Khan** | Logistic | Cauchy | GEV | Burr | Burr |
| **Kohat** | Cauchy | GEV | Burr | Burr | Cauchy |
| **Dir** | Cauchy | Burr | Johnson SB | Johnson SB | Burr |
| **Drosh** | Cauchy | Burr | LP-3 | Johnson SB | Burr |
| **Kakul** | Logistic | Johnson SB | LN(3P) | LN(3P) | Johnson SB |
| **Parachinar** | G. Pareto | GEV | Johnson SB | G. Pareto | Burr(4P) |
| **Peshawar** | Johnson SB | GEV | Johnson SB | Cauchy | Gamma |
| **Risalpur** | LP-3 | LN(3P) | Burr(4P) | Cauchy | LP-3 |
| **Saidu Sharif** | LN(3P) | Burr | Johnson SB | Johnson SB | Burr |
