# Supplementary material for: Design and analysis of statistical probability distribution and non-parametric trend analysis for reference evapotranspiration
Source: PeerJ. 2021 Jun 18;9:e11597. doi: 10.7717/peerj.11597 (PMC8216168; doi:10.7717/peerj.11597)
Supplement: Supplemental Information 5 [file peerj-09-11597-s005.docx]

| **Stations** | **Winter** | **Spring** | **Dry Summer** | **Monsoon** | **Autumn** |
| --- | --- | --- | --- | --- | --- |
| **Balakot** | Burr | Burr | Gamma(3P) | GEV | Burr(4P) |
| **Cherat** | Cauchy | G.Gamma | Logistic | Gumbel Max | Burr |
| **Chitral** | G.Gamma(4P) | Johnson SB | GEV | LP-3 | GEV |
| **DI Khan** | Burr | Cauchy | Rayleigh | Gumbel Max | Logistic |
| **Kohat** | Cauchy | G. Pareto | LN(3P) | GEV | Cauchy |
| **Dir** | Cauchy | GEV | Gumbel Min | LP-3 | Burr |
| **Drosh** | Cauchy | GEV | Johnson SB | Johnson SB | Gumbel Max |
| **Kakul** | Burr | Gamma | LP-3 | GEV | GEV |
| **Parachinar** | Johnson SB | GEV | GEV | G. Pareto | LP-3 |
| **Peshawar** | Johnson SB | GEV | GEV | Cauchy | Burr |
| **Risalpur** | Gumbel Max | Weibull | Cauchy | Cauchy | Weibull |
| **Saidu Sharif** | Cauchy | GEV | Gumbel Min | LP-3 | Burr(4P) |
